# GIPSy2: high-performance and scalable genomic island prediction software

**DOI:** 10.1038/s41598-026-53034-0

**Published:** 2026-06-05

**Authors:** Diego Lucas Neres Rodrigues, Pedro Alexandre Sodrzeieski, Doglas Parise, Ana Maria Benko-Iseppon, Vasco Azevedo, Siomar de Castro Soares, Flavia Figueira Aburjaile

**Affiliations:** 1 Department of Preventive Veterinary Medicine, Federal University of Minas Gerais, Belo Horizonte, Minas Gerais 31270-901 Brazil; 2 Department of Genetics, Federal University of Pernambuco, Recife, Pernambuco 50670-901 Brazil; 3 Department of Genetics, Ecology and Evolution, Federal University of Minas Gerais, Belo Horizonte, Minas Gerais 31270-901 Brazil; 4 Department of Microbiology, Immunology and Parasitology, Federal University of Triângulo Mineiro, Uberaba, Minas Gerais 38025-180 Brazil

**Keywords:** Mobilome, Horizontal gene transfer, Pathogenomics, Bioinformatics, Statistical predictions, Computational biology and bioinformatics, Genetics, Microbiology

## Abstract

Dealing with genomic mobility is a complex task for current predictors. With an increasing number of sequencing genomes, there is a constant demand for software that can handle multiple inputs. Considering this, we present the Genomic Island Prediction Software 2 (GIPSy2), a new version of well-established software for predicting bacterial genomic islands and mobilome. Statistical methods were used to provide the values associated with each prediction, such as Fisher’s exact test, Support vector machine, and Logistic regression. The new version also improves scalability, allowing the simultaneous analysis of multiple genomes, and provides structured outputs to facilitate interpretation and reproducibility. Comparative analyses show that GIPSy2 achieves performance comparable to the original version under default settings, while offering increased flexibility through user-defined parameterization. These improvements make GIPSy2 a versatile tool for genomic island prediction across diverse bacterial datasets. GIPSy2 is currently available on Zenodo repository at https://zenodo.org/doi/10.5281/zenodo.10222587.

**Supplementary Information:**

The online version contains supplementary material available at 10.1038/s41598-026-53034-0.

## Introduction

An extraordinary amount of data can be extracted from a genome because of its information-bearing properties, whether in terms of sequence or structure^1^. However, there is still a gap between obtaining and analysing genomic data. This is because, by comparative standards, although sequencing technologies have advanced at a brisk rate, investment in human resources to produce tools capable of handling extremely large datasets is still limited. However, new methods for detecting genetic sequence patterns and features have been developed and applied over time^2^.

One feature that is consistently present in bacterial genomes is the presence of genomic islands. They are contiguous sequence fragments > 10 kb in length and can contain up to 200 kb^3^. The interior of a genomic island may contain genes related to evolutionary processes of interest^4^. In pathogens, these elements are known to carry factors related to virulence, such as effectors, toxins, genes related to pili, motility, and adhesion, and are classified as pathogenic islands^5^. Furthermore, depending on the classification of the factors contained within it, a genomic island can be grouped into certain types, the most commonly used being antimicrobial resistance islands, islands of factors directed at symbiosis, and islands carrying factors for the biosynthesis of secondary metabolites^6,7^. Each type of island contains a significant number of factors related to its biological function, providing an adaptive advantage to bacterial cells or new capabilities for dealing with specific substrates^7^.

In addition to their adaptive properties, genomic islands are important for the distribution of gene content. Owing to their intrinsic characteristics, such as the presence of insertion sequences and repeats, islands are considered genomic hotspots for the insertion of sequences obtained by horizontal gene transfer (HGT)^8^. It is also possible for an entire island to be transferred during the transformation process^4^.

This fact, combined with the adaptive properties related to the acquisition of genes that promote advantages during selection processes, makes the study of bacterial genomic islands an ever-relevant topic but one of the particular concerns in light of the growing number of hyper-virulent and multi-resistant bacterial isolates^9,10^. Many phenotypic characteristics related to virulence and resistance are partially associated with known gene and molecular factor profiles. Generally, these factors have a constant potential for horizontal transfer and genetic fixation because of their niche-specific advantages; therefore, there is a well-defined relationship between pathogenicity and the presence of genomic islands in some species^11^.

Once genomic islands arise from HGT, one of their main characteristics is that they have molecular patterns that differ from other regions of the genome^12,13^. The coding sequences (CDS) within the islands have a distinct GC content bias compared to the regions present in other parts of the genome. Furthermore, these regions are mostly flanked by tRNAs, which allow the assignment of exogenous sequences by 3’-end insertions, and finally, genomic islands are also known hotspots for insertion sequences and tandem repeats^4,14^. Considering these characteristics, some programs have been developed to search for specific patterns of genomic islands and indicate their presence and location within the genome of a bacterial dataset^15^. Among the available programs, the Genomic Island Prediction Software (GIPSy)^16^, published in 2016, is considered one of the gold standards in terms of predictive accuracy^14,17^. However, regarding the scalability, the bottleneck of this tool must be addressed.

In this study, we present GIPSy2, an update of the previous version. The acronym GIPSy is retained for consistency with the previously published version of the software and its established use in the literature. This update aims to improve the scalability and reproducibility of the program as well as the reliability of the results obtained through statistical validation. This is in addition to maintaining previous features, such as predicting different classes of genomic islands and presenting itself as a standalone program with low computational consumption.

## Results

After implementing the new version of GIPSy, comparative analyses were conducted to evaluate the performance of the enhanced pipeline.

### Resistance Islands (REI) in *A. baumannii* AYE

**Fig. 1:**
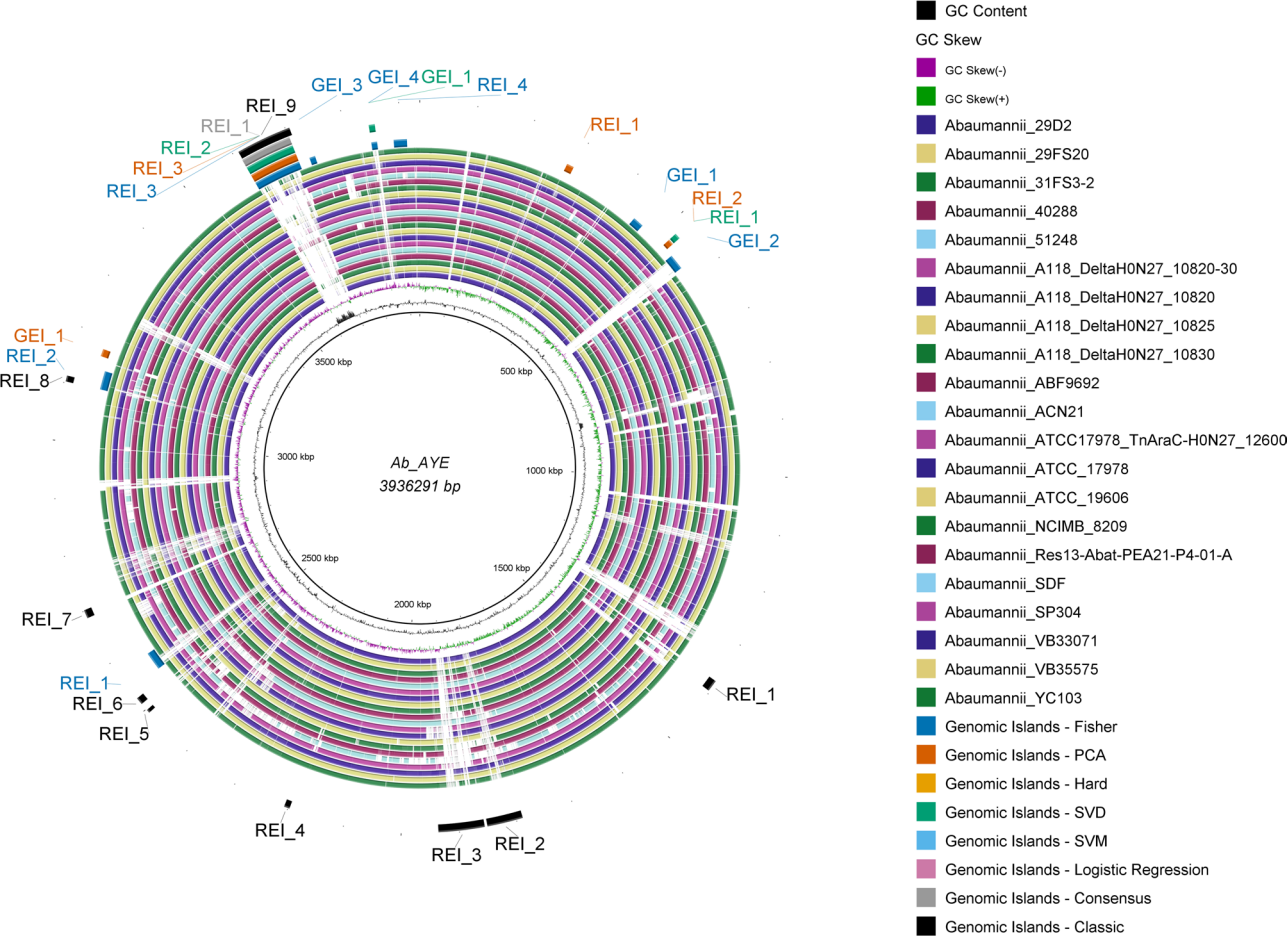
BLAST Atlas representing the comparison against *A. baumannii* AYE and other genomes of the same species. The first two rings in the center represent the GC content and the GC skew respectively. Each subsequent ring represents a genome used as a comparison following the order on the right side of the picture. Blank gaps represent regions present in the analyzed genome and absent in the genome representing the ring. The very last rings are representing the predicted genomic islands for each method deployed: blue—Fisher’s exact test; orange—normalized PCA eigenvalues; yellow—hard method; green—normalized SVD eigenvalues; sky blue—SVM classifier; purple—logistic regression classifier; gray represents islands predicted by at least three of the previous methods; black—GIPSy1 predictions.

As a result of the resistance islands prediction of *A. baumannii* AYE, GIPSy2 successfully predicted the so-called AbaR1 resistance island (Fig. 1), a singular genomic fragment of about 90 Kbp in length with about 93 coding sequences, 44% of which were classified as resistance-related proteins against at least one class of antimicrobial^18^ (see Supplementary table 1 and Supplementary table 2). This island was named REI_9 by GIPSy1 prediction and was the most important result for the strain in the context of this work, since it is the best documented island for the strain^18,19^.

In contrast, GIPSy2 predicted nine exclusive islands, that is, without any overlap between them and any other island predicted by GIPSy1. On the other hand, GIPSy1 predicts seven unique islands.

The islands predicted exclusively by GIPSy1 were evaluated individually for basic elements for island prediction (BEIP) content. Given the prevalence of BEIPs throughout the genome on average, a low relative presence of BEIPs was observed on all islands predicted exclusively by GIPSy1. In particular, a low presence of mobilome-related elements was observed.

Currently, BEIP content plays a significant role in predicting the position and size of islands. Therefore, it is expected that genomic island regions will have a higher proportion of these elements than the rest of the genome (see the Proksee session in Supporting Material (1). This, combined with the implementation of the consensus method, validated the genetic content of the island and its presence more reliably.

### Metabolic Islands (MEI) in *B. pseudomallei* K96243

**Fig. 2:**
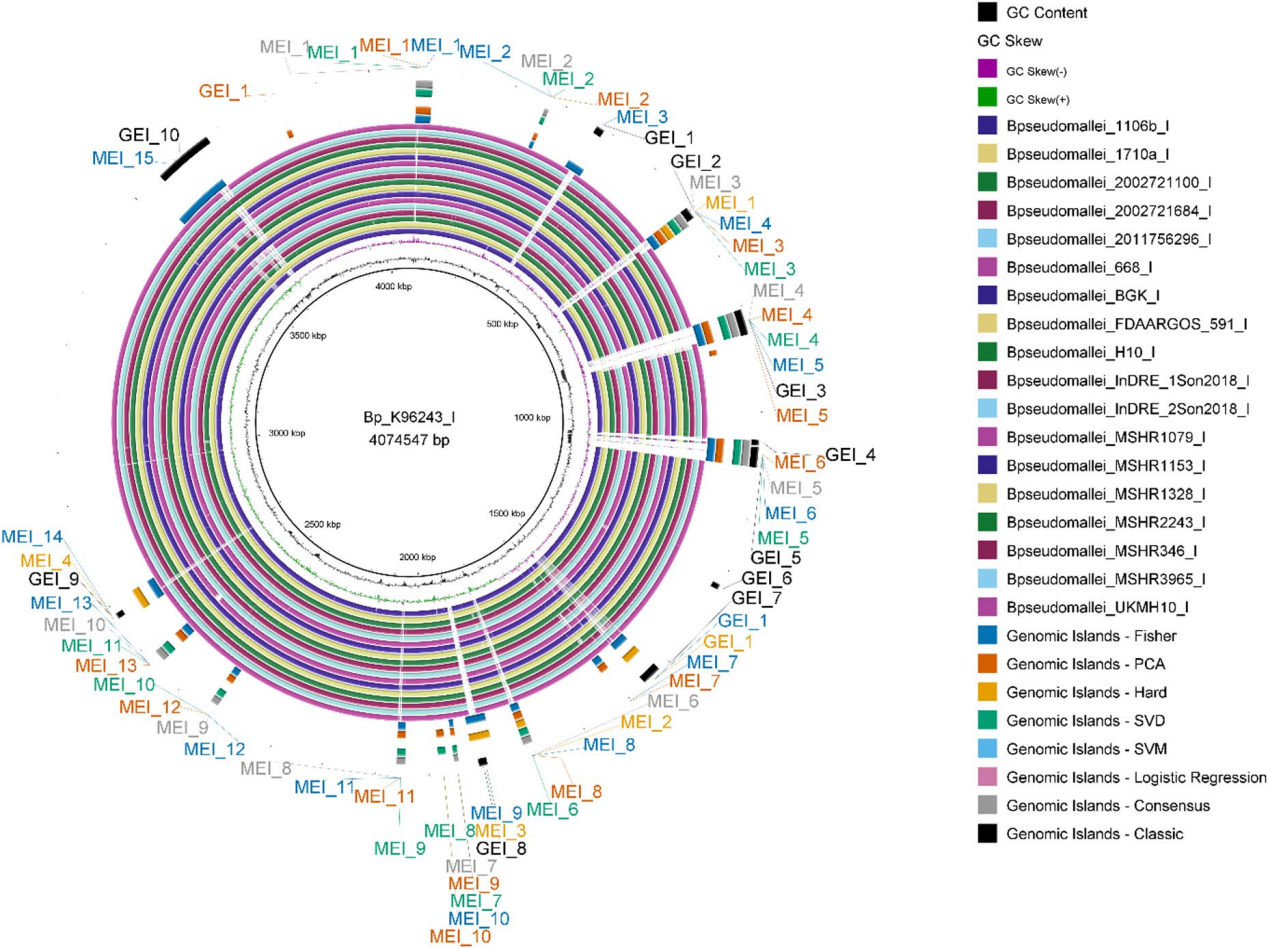
BLAST Atlas representing the comparison against *B. pseudomallei* K96243 chromosome I and other genomes of the same species. The first two rings in the center represent the GC content and the GC skew respectively. Each subsequent ring represents a genome used as a comparison following the order on the right side of the picture. Blank gaps represent regions present in the analyzed genome and absent in the genome representing the ring. The very last rings are representing the predicted genomic islands for each method deployed: blue—Fisher’s exact test; orange—normalized PCA eigenvalues; yellow—hard method; green—normalized SVD eigenvalues; sky blue—SVM classifier; purple—logistic regression classifier; gray represents islands predicted by at least three of the previous methods; black—GIPSy1 predictions.

The presence of two chromosomes is a characteristic of *Burkholderia pseudomallei*^20,21^; therefore, both chromosomes were analysed individually. On the first chromosome, eight genomic islands were predicted to overlap with the regions obtained by GIPSy1 (Fig. 2). Thus, virtually all reference regions predicted by GIPSy1 were also found using at least one GIPSy2 method. The only reference region not predicted by GIPSy2 was Genomic Island 6 (GEI_6) using the classical methods. However, when the content in this region (1,330,288..1340212) was examined, no significant content of BEIPs was found within the putative region or in regions adjacent to it (see the Proksee section in Supporting Material 2).

Moreover, 12 new putative regions were predicted exclusively using at least one of the GIPSy2 methods (see Supplementary table 1 e Supplementary table 3).

**Fig. 3:**
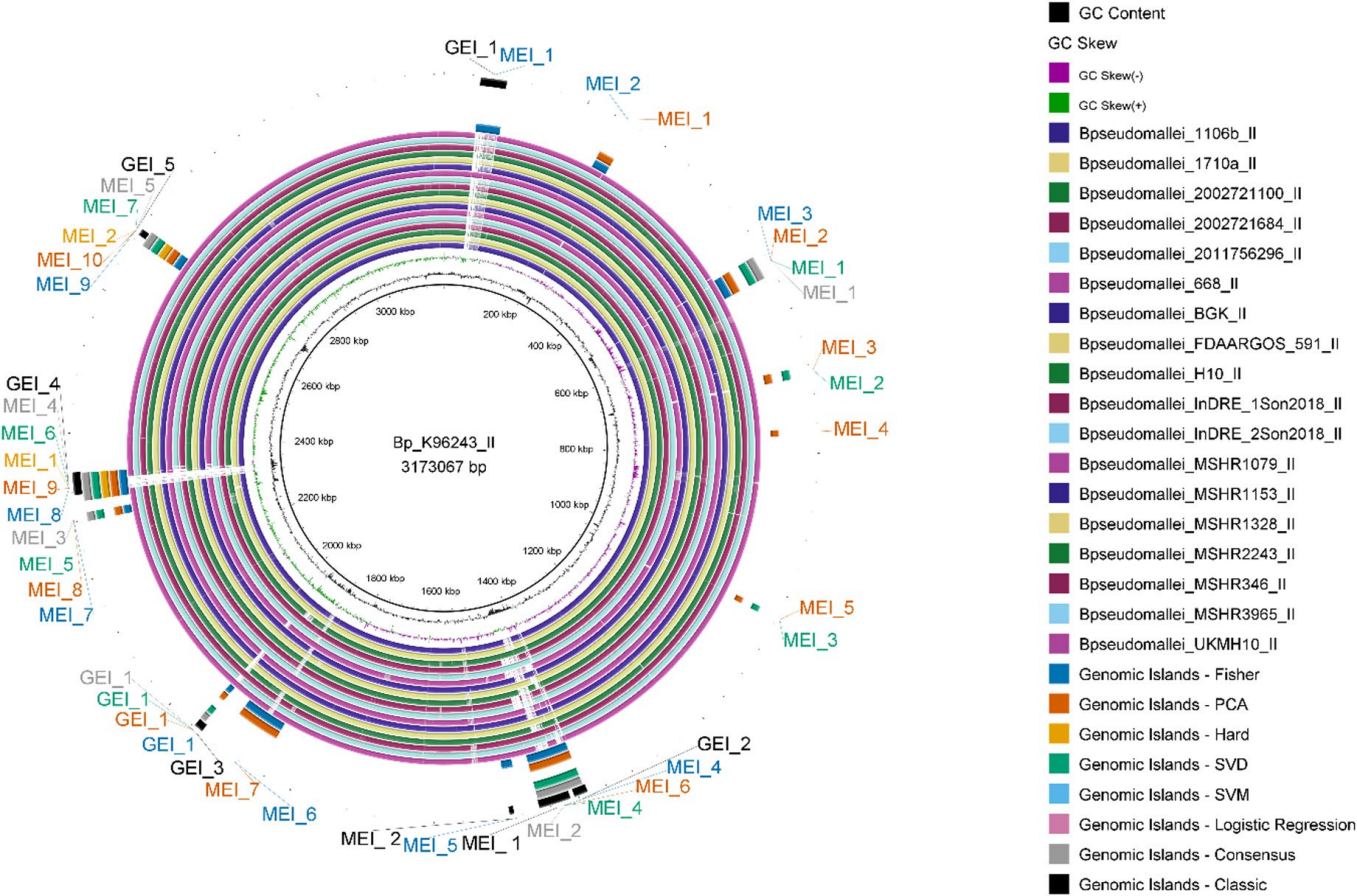
BLAST Atlas representing the comparison against *B. pseudomallei* K96243 chromosome II and other genomes of the same species. The first two rings in the center represent the GC content and the GC skew respectively. Each subsequent ring represents a genome used as a comparison following the order on the right side of the picture. Blank gaps represent regions present in the analyzed genome and absent in the genome representing the ring. The very last rings are representing the predicted genomic islands for each method deployed: blue—Fisher’s exact test; orange—normalized PCA eigenvalues; yellow—hard method; green—normalized SVD eigenvalues; sky blue—SVM classifier; purple—logistic regression classifier; gray represents islands predicted by at least three of the previous methods; black—GIPSy1 predictions.

On the second and smallest chromosomes, six genomic islands were predicted to overlap with the regions obtained using GIPSy1 (Fig. 3). Therefore, all reference regions were predicted using at least one of the GIPSy2 methods. In addition, seven new putative regions were predicted exclusively using at least one of the GIPSy2 methods (see the Proksee section in Supporting Material 3).

The contents of the predicted islands for chromosome II can be found in Supplementary table 1 and Supplementary table 1.

### Pathogenicity Islands (PAI) in *E. coli* CFT073

**Fig. 4:**
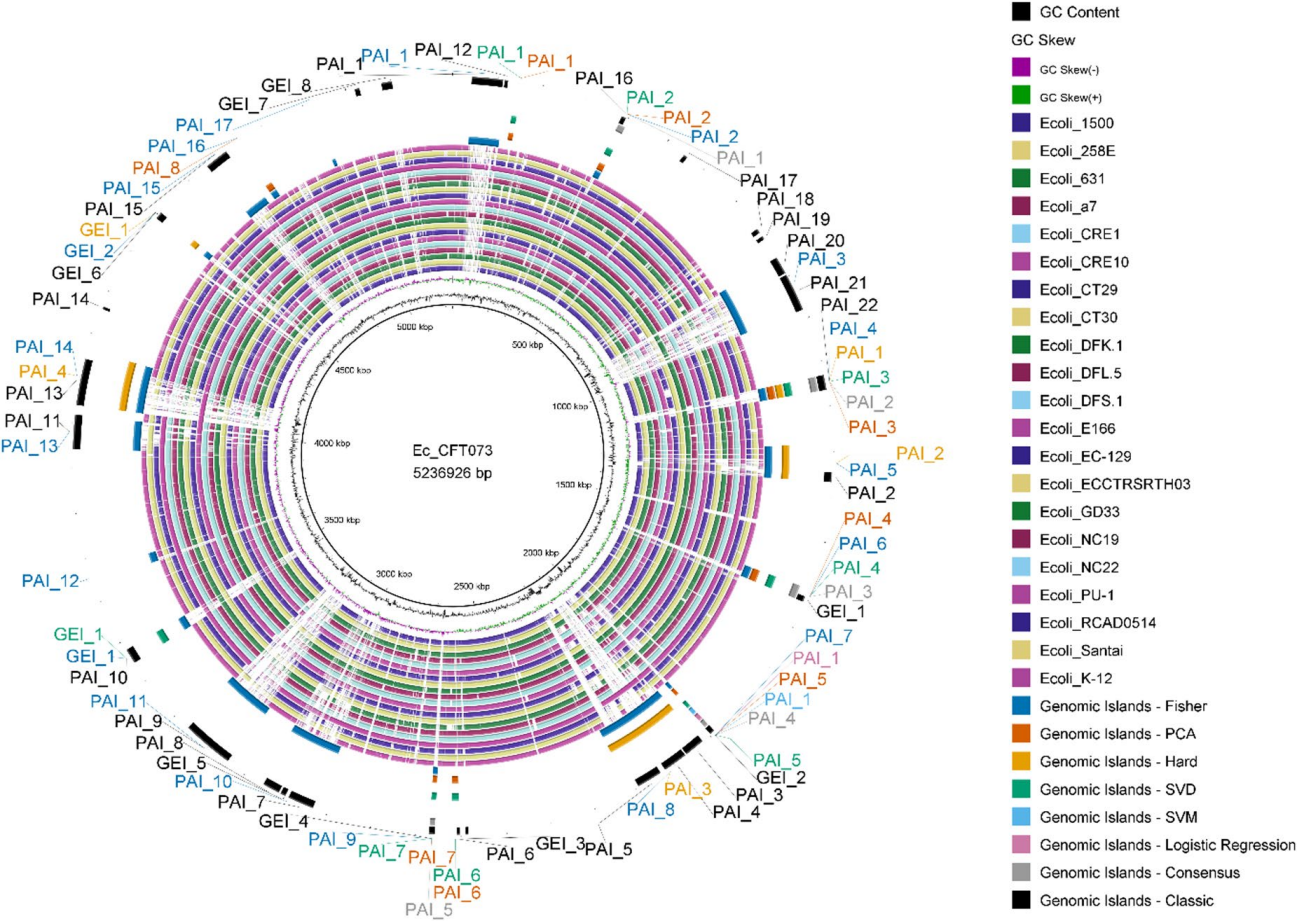
BLAST Atlas representing the comparison against *E. coli* CFT073 and other genomes of the same species. The first two rings in the center represent the GC content and the GC skew respectively. Each subsequent ring represents a genome used as a comparison following the order on the right side of the picture. Blank gaps represent regions present in the analyzed genome and absent in the genome representing the ring. The very last rings are representing the predicted genomic islands for each method deployed: blue—Fisher’s exact test; orange—normalized PCA eigenvalues; yellow—hard method; green—normalized SVD eigenvalues; sky blue—SVM classifier; purple—logistic regression classifier; gray represents islands predicted by at least three of the previous methods; black—GIPSy1 predictions.

For pathogenicity island prediction analysis in *E. coli* CFT073, 17 islands were predicted to overlap with putative regions resulting from GIPSy1 analysis (Fig. 4). A significant number of fragments was predicted using only Fisher’s exact test or hard methods–10 islands.

Four islands were predicted only by GIPSy2 and seven islands were predicted only by GIPSy1. Considering the GIs predicted only in GIPSy1, only regions with tangential PAI_5, PAI_6, and GEI_8 had some mobilome elements related to the sequence (see Proksee section in Supporting Material 4). None of the other putative islands identified exclusively by GIPSy1 contained BEIPs. In contrast, PAI_16 and PAI_17, found exclusively on GIPSy2 using Fisher and PCA methods, have a significant number of tRNA genes inside the island but no mobilome elements.

The content of the predicted islands for *E. coli* CFT073 are shown in Supplementary table 1 and Supplementary table 5.

### Symbiotic Islands (SYI) in *M. japonicum* MAFF 303,099

**Fig. 5:**
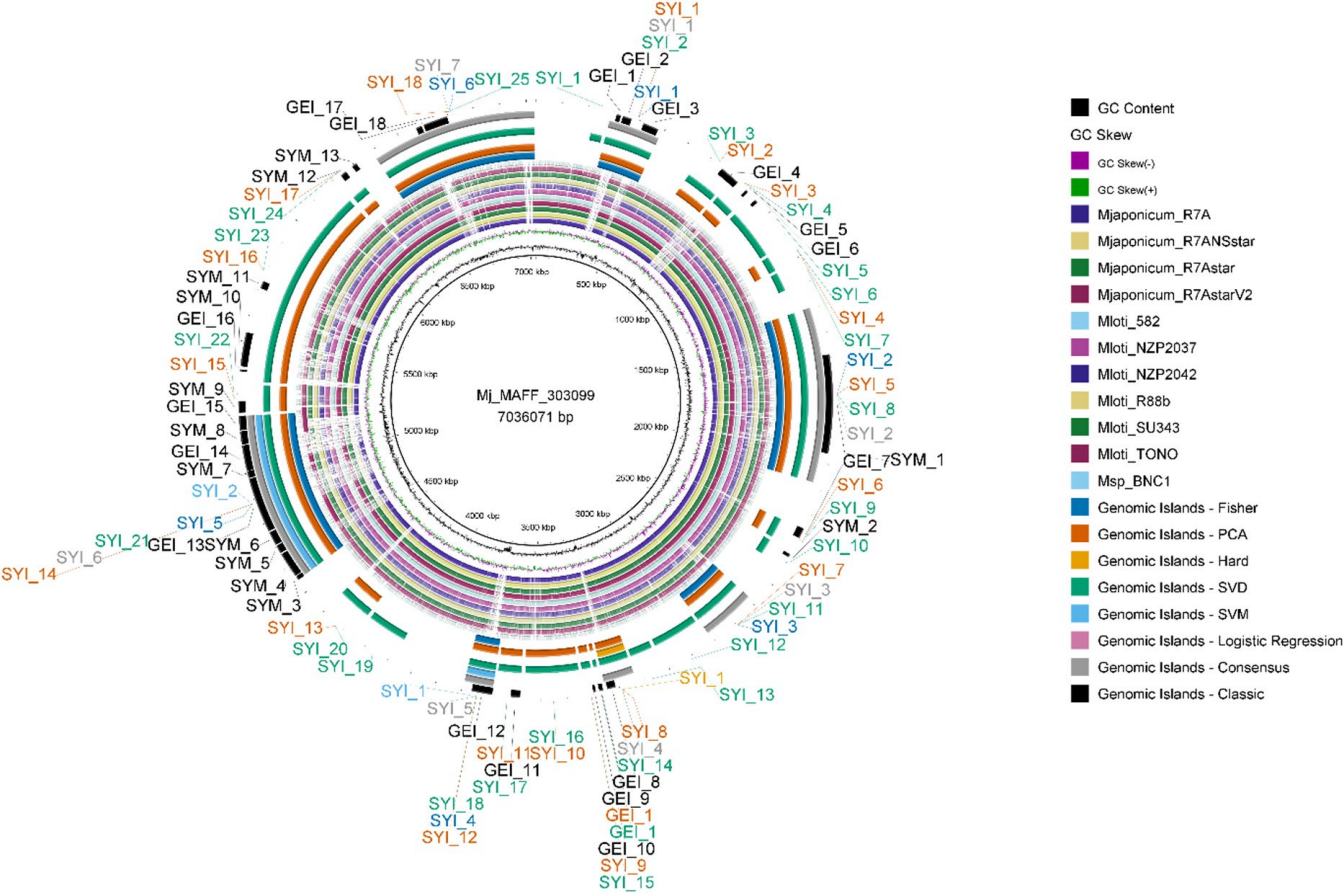
BLAST Atlas representing the comparison of *M. japonicum* MAFF 303,099 against other genomes of the same species. The first two rings in the center represent the GC content and the GC skew respectively. Each subsequent ring represents a genome used as a comparison following the order on the right side of the picture. Blank gaps represent regions present in the analyzed genome and absent in the genome representing the ring. The very last rings are representing the predicted genomic islands for each method deployed. The *Mesorhizobium sp.* BNC1 genome was used as reference for genomic island prediction: blue—Fisher’s exact test; orange—normalized PCA eigenvalues; yellow—hard method; green—normalized SVD eigenvalues; sky blue—SVM classifier; purple—logistic regression classifier; gray represents islands predicted by at least three of the previous methods; black—GIPSy1 predictions.

As a first result of the prediction of genomic islands in *M. japonicum* MAFF 303,099, a lower number of islands was predicted by GIPSy2 (eight on average) than GIPSy1 (31 in total) (Fig. 5). However, the regions predicted by the new version tend to be larger and have a total overlap of several islands simultaneously, as in the case of the SYM_6 island predicted by the GIPSy2 consensus method, which overlaps with nine islands predicted by GIPSy1. Using the consensus method, a higher prevalence of mobilome elements was observed in the BEIP content of each island, especially within SYM_6 (see Proksee session in Supporting material 5, Supplementary table 1 and Supplementary table 6).

**Fig. 6:**
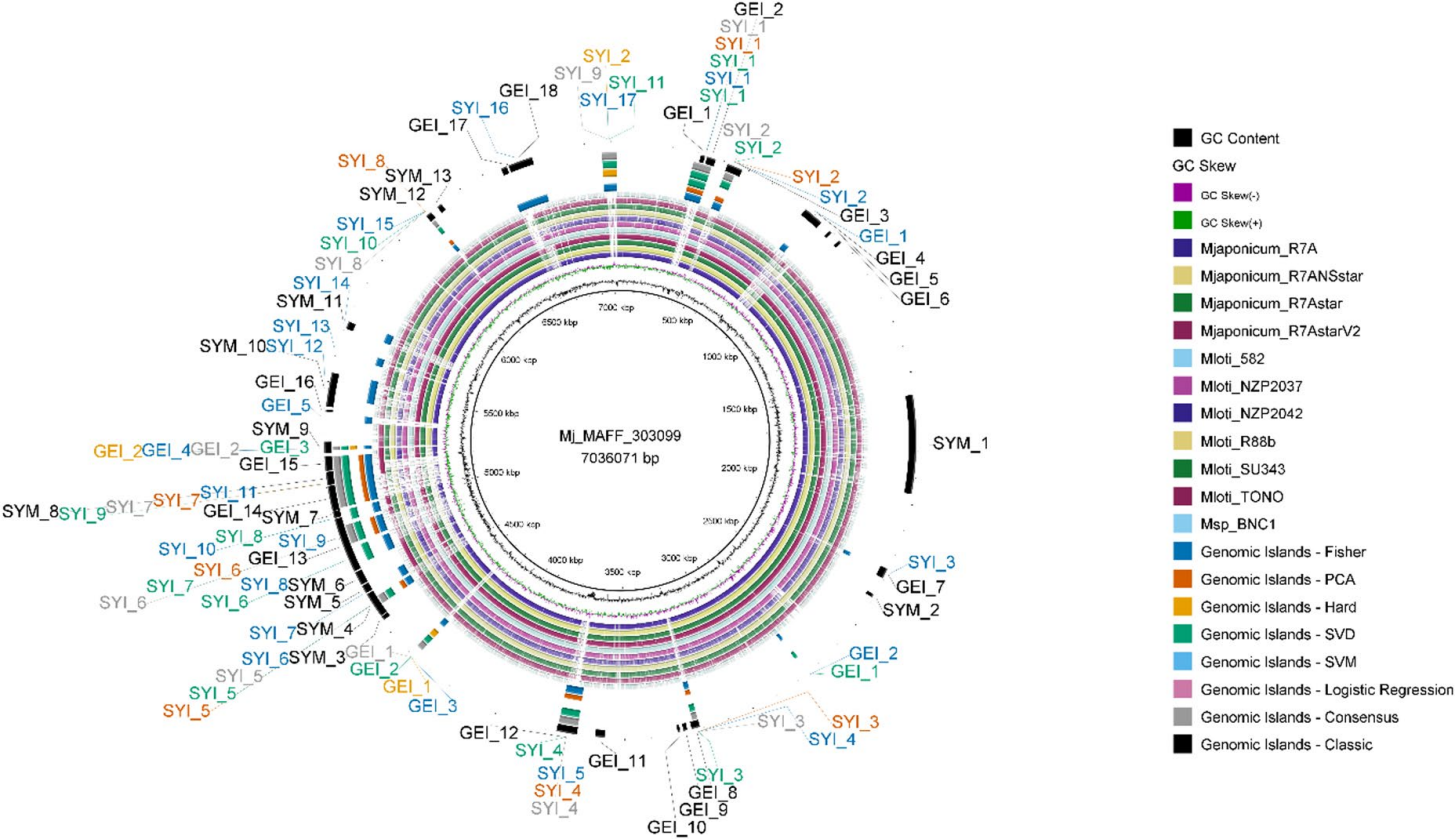
BLAST Atlas representing the comparison of *M. japonicum* MAFF 303,099 against other genomes of the same species. The first two rings in the center represent the GC content and the GC skew respectively. Each subsequent ring represents a genome used as a comparison following the order on the right side of the picture. Blank gaps represent regions present in the analyzed genome and absent in the genome representing the ring. The very last rings are representing the predicted genomic islands for each method deployed: blue—Fisher’s exact test; orange—normalized PCA eigenvalues; yellow—hard method; green—normalized SVD eigenvalues; sky blue—SVM classifier; purple—logistic regression classifier; gray represents islands predicted by at least three of the previous methods; black—GIPSy1 predictions. *Mesorhizobium sp.* BNC1, *M. japonicum* R7Astar, *Mesorhizobium loti* R88b and *M. loti* TONO genomes were used as reference for genomic island prediction.

Owing to the greater length of islands generated by the new version, the analysis was repeated considering an analysis with multiple references compared to lineages closer to the query strain. The results of the new analysis are shown in Fig. 6. From this result, the overall final size of the predicted islands was markedly reduced; however, when looking at the BEIP distribution, there is a higher proportional concentration of elements in the newly predicted islands (see proksse session in Supporting material 6, Supplementary table 1 and Supplementary table 7).

### Performance analysis

**Fig. 7:**
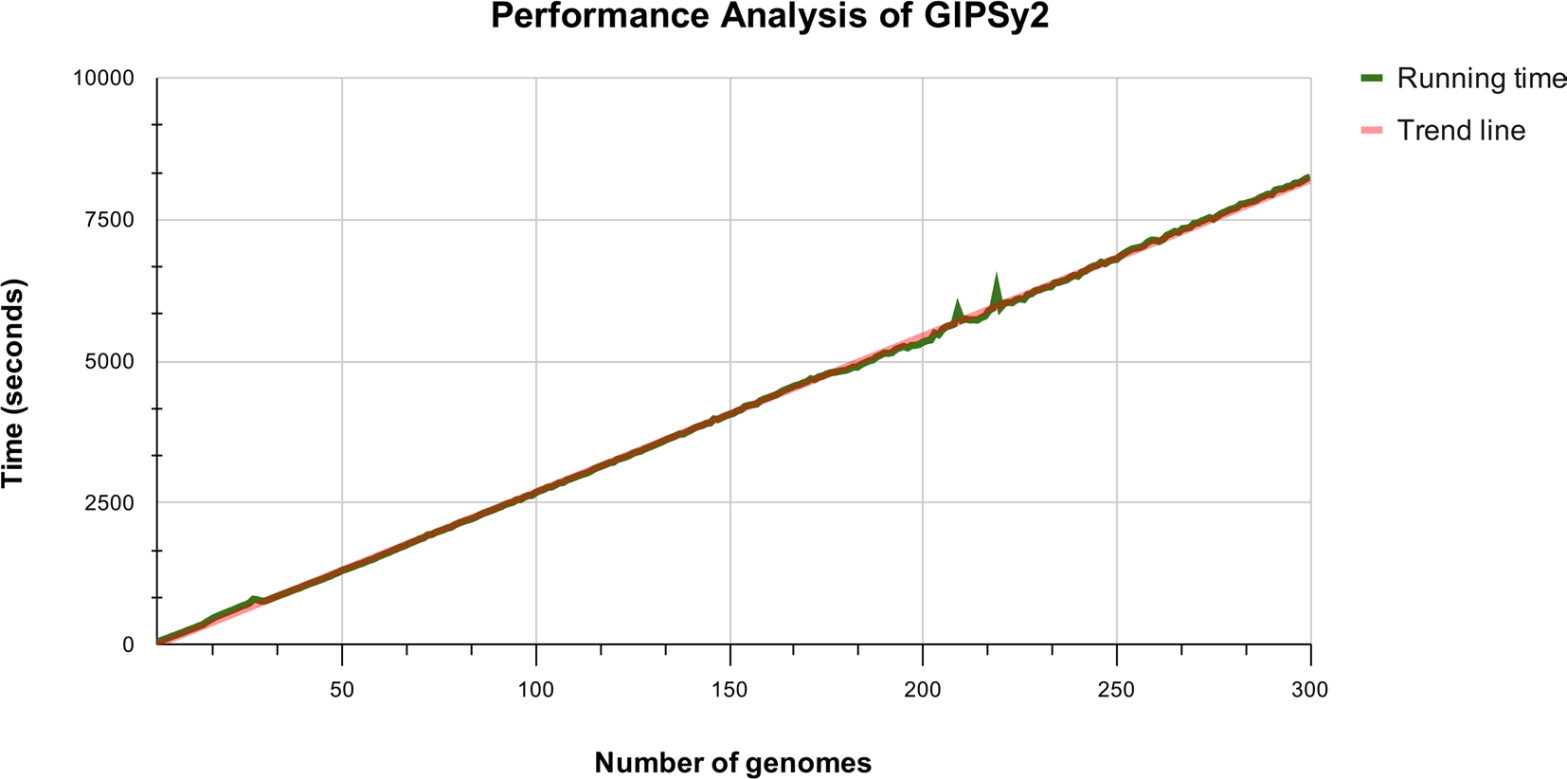
Line graph representing the time consumption of GIPSy2 analysis considering consecutive increases in the sample.

As a result of the performance analysis, the graph represented by Fig. 7 shows the time required to complete the analysis of the entire dataset used as input.

The graph shows that the complexity of GIPSy2 follows a linear progression that obeys the function *f(x) =(27.534× n) -31.249* and has a reference value of R^2^ = 0.9997. GIPSy2 required 2 h 17 min and 55 s to analyse 300 genomes of *E. coli* strains. Using the previous function, it can be inferred that analysing 1,000 *E. coli* genomes with GIPSy2 using the same setup would take approximately 8 h.

Table 1 shows the results of the precision tests for GIPSy1, GIPSy2 using the newly described Fisher’s method, and IslandViewer. The best ad hoc parameterisation considered a minimum island size of 30 kb and a minimum distance between two islands (namely, step size) of 4.5 Kbp, which resulted in a given outcome. The results of this study indicate that, under default parameterization, GIPSy1 exhibited superior performance to GIPSy2 in the accuracy calculation by 0.3%. In contrast, when the correct parameterization is employed, as represented by the ad hoc model, the accuracy of GIPSy2 surpasses that of GIPSy1 by approximately 6%. Furthermore, GIPSy2 demonstrates low relative values of false positives and false negatives, even in comparison to the other methods considered. Interestingly, we can observe the single result where GIPSy 1 outperforms GIPSy 2 in terms of accuracy. This occurs during the prediction process because GIPSy 2 proved to be more permissive of false positive results during this test, predicting larger and less followed islands. However, GIPSy 2 with the default parameterization prediction proved to be efficient in avoiding false negatives, being the second model with the lowest number of false negatives and the one with the highest sensitivity value.

**Table 1:** Main precision test results for genomic island prediction.

Software	Accuracy	Sensitivity	Specificity	False positive	False negative
GIPSy1	0.718	0.256	0.805	811	587
GIPSy2 (default)	0.715	0.307	0.791	869	546
GIPSy2 (ad hoc)	0.778	0.304	0.868	547	549
IslandViewer (Integrated)	0.745	0.359	0.817	767	505
IslandViewer (SIGI-HMM)	0.785	0.068	0.921	329	735
IslandViewer (IslandPick)	0.786	0.100	0.916	347	710
IslandViewer (DIMOB)	0.787	0.176	0.905	384	650

**Fig. 8:**
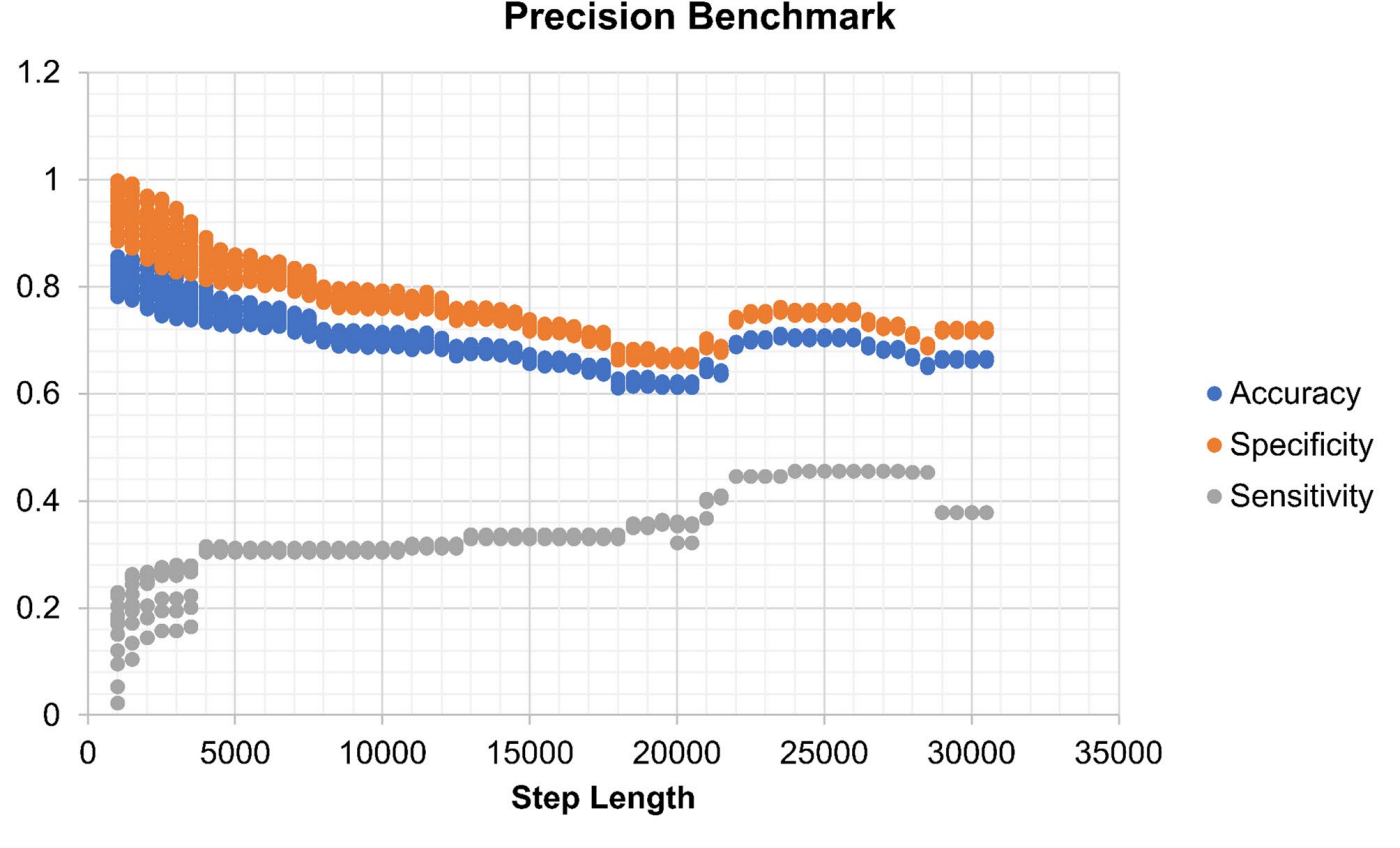
Dispersion graph representing the results of precision tests performed using GIPSy2. Step length means the space between two sequential islands in pairs of bases.

Figure 8 displays the results of the accuracy analysis based on the step size between upstream and downstream islands. The results indicate that accuracy and specificity decrease as sensitivity increases. For the raw analysis results, please refer to Supplementary table 8.

## Discussion

The new GIPSy2 algorithm highlights the scalability and performance required by genomic researchers^22^. With the increasing demand for genomic analysis tools, tools capable of handling multiple bacterial genomes simultaneously in a reasonable amount of time have already proven extremely useful in scientific routines^23^. In addition, the features used by GIPSy2, including compositional bias, codon usage deviation, mobilome-associated genes, and tRNA flanking regions, are widely recognized in the literature as hallmarks of genomic islands and horizontal gene transfer^4,8,16,24^. These signals have been extensively explored in computational methods for genomic island detection, supporting their biological relevance and robustness across bacterial species.

For comparison and to validate the new results, the analyses presented in the article that published the first version of GIPSy were replicated in this study. However, the results of these predictions were not identical representing the impacts of the methodological changes in the overall pipeline. A key reason for this divergence between the results is the change in the criterion for capturing the regions in Step 9. Allowing the pipeline to add genes to a region based on its deviation from the GC and not just its absence in a reference allowed an increase in the accuracy of capturing putative regions, especially considering that this is one of the main features observed in genomic islands^8,25^. It also dissociates and reduces the risk of not capturing a region by comparing it with an inappropriate reference. Nevertheless, a previous work of Bohlin et al.^26^ have shown little or no relationship between intragenomic variation in GC content and the presence of genomic islands, although, notably, GC deviation is used as an indicator of position relative to an island and alone does not reproduce the correct prediction of a genomic island.

To discuss the comparison results it is important to highlight: comparison of genomic island predictions between different methods is inherently challenging due to variability in predicted boundaries, segmentation, and resolution. Genomic islands are not discrete entities with universally defined limits, and their identification depends on methodological assumptions and parameter settings^4,11,15,27^. As a result, overlap-based comparisons may be highly sensitive to arbitrary thresholds and may not accurately reflect biological agreement between predictions.

By applying GIPSy to predict the islands in *A. baumannii* AYE, the new workflow correctly predicted the reference island for strain REI_9. This region was described and confirmed in previous work and preliminary analyses of GIPSy1^18,28^, and this island was confirmed using three different statistical models of GIPSy2. However, different putative regions were predicted for GIPSy1 and GIPSy2 (Fig. 1). By examining these regions in the Proksee session, it was possible to determine that GIPSy2 most of them contained an accumulation of tRNAs and mobilome elements, which increased the final statistical score for these positions. However, by looking at the generated BLAST atlas, it is also possible to see that the absence of most of these islands in comparative genomes is a strong indication of genomic mobility, as already described in the literature^4,29^. Notably, the best prediction method for this strain was the Fisher method, which correctly predicted not only the reference island but also the islands with a high BEIP signature. Furthermore, changing the reference used may result in a new set of islands that can be predicted, resulting in greater coverage of the GIPSy1 islands. However, by evaluating the BEIP content of the exclusive GIPSy1 islands, it was not possible to determine the accuracy of the prediction, as the content of BEIPs is less than the minimum standard established by GIPSy2.

By analysing the results for both chromosomes of *B. pseudomallei* K96243, it was possible to notice a greater linearity between the results, that is there was a greater overlap between the islands of GIPSy1 and GIPSy2. However, GIPSy2 had the highest number of predicted islands. When observing the results of previous studies that analysed the genomic mobility of this strain of *B. pseudomallei*^30^, it was observed that GIPSy2 did not correctly predict the island called GEI_6 by version 1 for chromosome 1; however, when observing the content of this island using Proksee, it was not possible to observe any content of BEIPs inside or near to it. This indicates either a false positive confirmed by its presence in most of the comparative genomes observed or a positive result that could not be captured by GIPSy2 owing to peculiar characteristics, such as absence of GC bias or structural features, i.e., tRNA sequences. Another study also verified the presence of genomic islands in *B. pseudomallei* and, in contrast, the number and position of the islands varies according to the strain chosen as a reference^21^. Another important fact is that considering the reference work, only GIPSy2 was able to correctly capture the MEI_2 island considered by the consensus method, as well as being able to capture the MEI_5 island without fragmenting it. One potential explanation for this phenomenon is the synergy between the utilization of reference information and the employment of the GC skew offset, which collectively fostered the identification of the region as a potential island in the updated pipeline.

As this isolate is one of the best described in terms of genomic mobility^31,32^, analysis of the *E. coli* strain CFT073 represents a comparative gold standard for genetically mobile elements related to pathogenicity. GIPSy2 was found to predict four unique islands, two of which were predicted exclusively by the Fisher method and two by the PCA-based method. When analysing these exclusive islands in the Proksee session, we observed that the PAI_1 island predicted by the PCA-based method and the PAI_16 island predicted by the Fisher method did not have content related to the mobilome, such as integrases and recombinases. However, they had a high number of CDS with deviations in codon usage, which is a strong indication of genetic mobility that justifies the prediction of these islands by new scores^33^. In contrast, the PAI_12 island predicted by Fisher’s method contains elements related to the mobilome and tRNAs of different qualities, which is important for predicting mobile genetic elements, especially genomic islands^34^. The PAI_17 island predicted by Fisher’s method, on the other hand, is surrounded by elements related to genetic mobility and contains some tRNAs as well as a complete ribosomal RNA operon. These sequences have already been reported in bacteriophage-mediated transformations^35^ and are evolutionary clues because higher numbers of rRNAs indicate higher replication rates^36^. As stated, among the various genomic island indicators, the presence of mobility elements, flanking tRNAs, and compositional bias are often strong predictors in horizontally acquired regions. In this context, the accumulation of multiple features in PAI_17 strengthens its prediction as a genomic island.

The analysis performed on *M. japonicum* MAFF 303,099 is relevant to show not only the linearity of GIPSy2 analysis but also the importance of choosing a good reference for genomic plasticity analyses, something that has been mentioned and discussed in the literature^15^. It also highlights the impact of the recently implemented multireference analysis. Considering only the results shown in Fig. 6, GIPSy2 predicts five unique islands using at least one of the applied methods. Of these, four islands (GEI_2, GEI_3, GEI_5, and SYI_17 by the PCA-based method) had a significant amount of BEIP in their contents, such as mobilome-related elements and genes with deviations in codon usage and were flanked or had tRNAs at their ends. In contrast, the SYI_13 island predicted by the PCA-based method did not have tRNAs on its flanks, but did have a large number of mobilome-related elements. Despite the greater divergence between GIPSy1 and 2, the analysis shown in Fig. 5 was able to reproduce with even greater reliability and linearity the reference island for the strain presented in the literature^37^, which was predicted by the Fisher method and henceforth called SYI_5. It can be claimed with a high degree of confidence that the most significant contribution to this achievement is the integration of the method based on multiple references. In such cases, the utilisation of supplementary variables to extract equivalent information serves to minimise the probability of error and enhance reliability. We can therefore say that the new GIPSy method is particularly effective in predicting genomic islands in the genomes of bacteria that have been little studied but which have a considerable number of genomes available. This is a major achievement when comparing both versions of GIPSy, since it is rather unique for this field.

Finally, accuracy tests applied to GIPSy2 revealed that specificity and accuracy decreased as sensitivity increased, a well-known and expected phenomenon^38^. Moreover, the accuracy values obtained by GIPSy2 with standard parameters are competitive when compared to GIPSy1 and IslandViewer and tend to be higher when considering the specific parameterisation used as a base for the study. However, this result must be evaluated with caution because the reference study for precision analysis has the limitation of only considering islands larger than 30 kb. Therefore, GIPSy2 predicted islands smaller than 30 kb, in which several strains showed deletions in these regions, as depicted in Fig. 4. It is important to note that the improved accuracy observed under the ad hoc parameterization is influenced by the alignment between the selected parameters and the constraints of the benchmark dataset. Specifically, the minimum island size of 30 kb reflects the resolution limit of the microarray-based approach used by Lloyd et al.^63^, which does not detect smaller genomic islands. As a result, regions below this threshold are excluded from evaluation, reducing the number of apparent false positives. Therefore, the observed increase in accuracy should be interpreted with caution and does not necessarily reflect a general improvement in predictive performance across datasets. Therefore, to obtain more accurate results, a definitive laboratory study of the genomic islands in a microbial model is necessary.

It is important to note that machine learning-based scores, such as those obtained from SVM and logistic regression, are derived from internally constructed labels and assumptions regarding genomic island absence in reference genomes. Therefore, these scores should be interpreted as complementary evidence within the integrative prediction framework.

Additionally, GIPSy2 relies on genomic features that are broadly conserved across bacterial species, such as compositional bias, codon usage deviation, and the presence of mobilome-associated elements. This allows the default parameterization to provide a general-purpose configuration applicable to diverse datasets. In practice, this reduces the need for species-specific parameter tuning, although adjustments may improve performance depending on genome architecture and evolutionary context. Certain parameters, such as minimum island length and gene distance thresholds, may influence prediction outcomes depending on genome structure and the specific biological question being addressed. Therefore, GIPSy2 was designed to allow user-defined parameterization, enabling adaptation to specific datasets or research objectives. Future studies evaluating parameter stability across a broader range of species would further improve the generalizability of the approach.

Furthermore, the previous study depended on a reference strain and its technique relied on the use of well-constructed probes. In other words, the obtained results reflect the ability of the programs to reproduce the experimental results; however, the precision values may differ depending on the limitations intrinsic to laboratory techniques. Given the above information, it is difficult to determine whether the false-negative and false-positive values of GIPSy2 are true prediction errors, especially given the number of BEIPs within the islands that are considered false predictions.

## Conclusion

GIPSy2 demonstrated performance comparable to its original version under default settings, and improved accuracy when parameterized for specific datasets, in addition to offering greater scalability and flexibility, and even more customisable by allowing the fine parameterisation of criteria not previously allowed, such as the size and minimum distance between islands. Furthermore, by providing a numerical value as a score at the end of the analysis, the result is more interpretable for the bioinformatics user community, allowing for better separation of putative genomic islands based on their content.

The predictions made by GIPSy2 were satisfactory for correctly inferring the position of the predicted islands for references that have already been well studied in terms of mobile factors in their genomes. In addition, the program’s complexity analysis provided a fitting function, indicating a low time cost for multi-genome analyses, making the analysis and interpretation of results from large datasets more accessible and less complex. Thus, GIPSy2 presents scalability and agility in the analysis of genomic plasticity, which is a major advancement in the scientific community.

Additionally, the GIPSy2 pipeline was designed and evaluated for its capacity to analyze bacterial genomes. Although the pipeline has the capacity to evaluate archaean and eukaryotic genomes, it is imperative to perform these analyses with a high degree of caution due to the ongoing need to validate this specific type of data. It has been demonstrated that genomic islands from organisms other than bacteria exhibit structural patterns that diverge from the main patterns sought by GIPSy. This divergence can eventually lead to the generation of anomalous results.

## Methods

Compared to GIPSy1, GIPSy2 introduces several key improvements. First, the pipeline was redesigned to support large-scale analyses, enabling the processing of hundreds to thousands of genomes with improved computational efficiency. Second, new statistical approaches, including machine learning methods and multivariate analyses, were incorporated to enhance the detection of genomic islands. Third, the integration of multiple genomic features—such as compositional bias, codon usage, mobilome-associated genes, and tRNA flanking regions—was expanded and refined. Finally, GIPSy2 provides increased flexibility through user-defined parameterization, allowing adaptation to different datasets and research objectives. Together, these improvements aim to enhance the robustness, scalability, and interpretability of genomic island prediction.

Unlike its first version, GIPSy 2 was developed in Python 3^39^. To function properly, the software takes advantage of two main dependencies: DIAMOND^40^ and Colombo/Sigi-HMM^41^. Specific databases containing resistance, virulence, symbiosis, and secondary metabolite biosynthesis factors were used to classify the islands resulting from initial analyses. Dependencies and databases were automatically downloaded in the first run of the program with functional parameters, and the presence of databases and dependencies was checked in subsequent rounds. To increase the agility and scalability of the analysis, GIPSy2 was designed to be executed entirely from the command line. In summary, the second version of GIPSy differs from the first in several methodological aspects. These include the incorporation of a module dedicated to the statistical analysis of the predictions of putative genomic islands; the utilization of matrix comparison methods to infer BEIPs in putative islands; the addition of a module that enables the analysis of multiple genomes simultaneously, applicable to both query and reference genomes; and a general update of the databases employed.

**Fig. 9:**
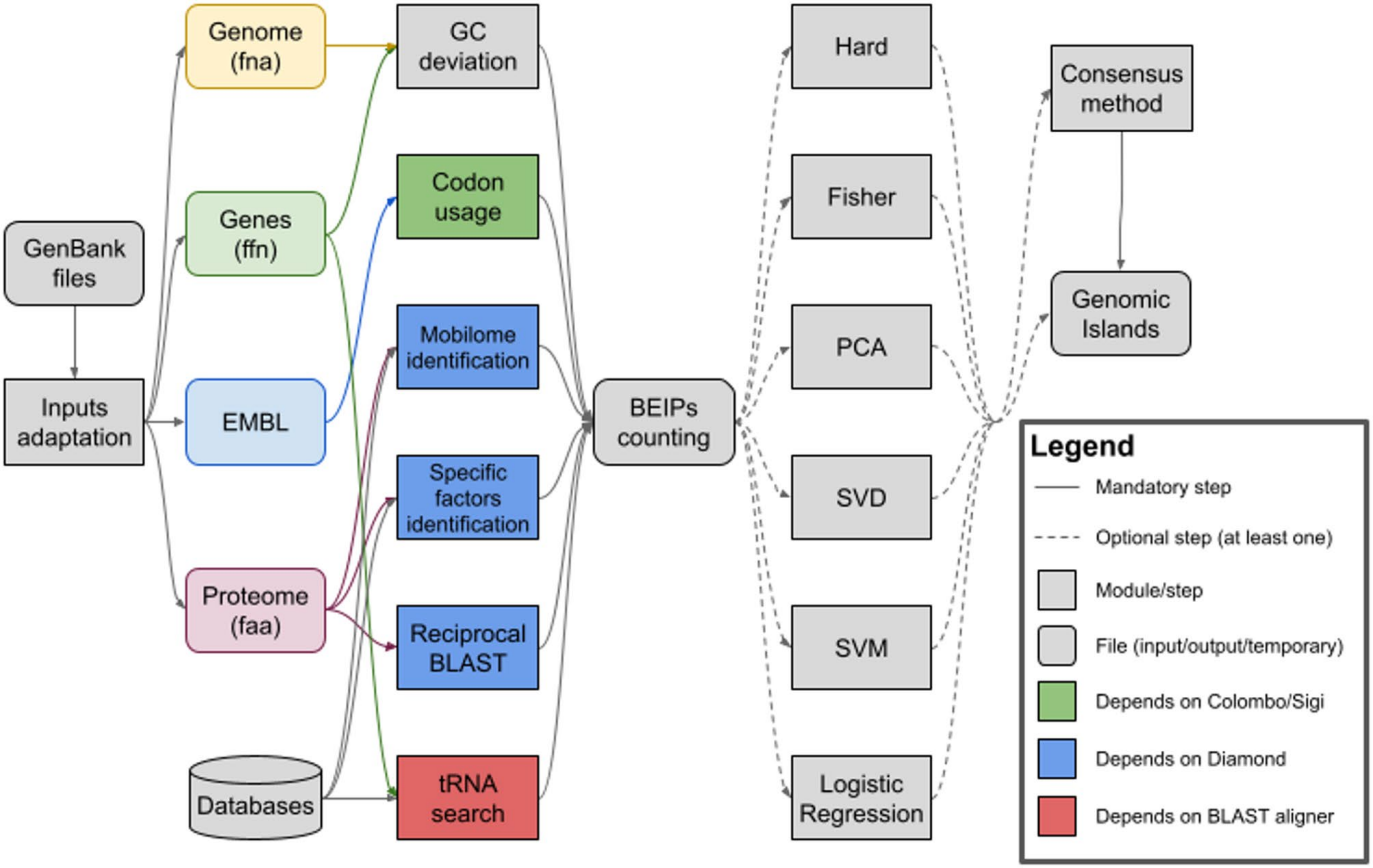
Chart that explains GIPSy2 data flow and all steps expected to be executed by the pipeline.

In general, the pipeline presented in Fig. 9 searches for BEIP. The following topics outline and clarify the steps taken by GIPSy 2.

### Step 1: input preparation

GIPSy 2 can handle files annotated using different methods, including the format available on the RefSeq platform; however, it is recommended that genomes be annotated in a standardised manner using the PROKKA pipeline^42^. The main input to the pipeline is GenBank files (gbk, gbf, and gbff). This type of file contains all structural information about the genome, which is essential for the next steps and dependencies. This information can be partitioned into (I) genome sequences, (II) amino acid sequences, (III) gene nucleotide sequences, and (IV) gene coordinates. It is possible to use GIPSy2 to analyze complete and incomplete genomes, as well as metagenome-assembled genomes, however, the user should ensure the quality and standardisation of the proposed inputs as well as the presence of the aforementioned basic information.

Therefore, the first step in the pipeline is to extract the data required for the dependencies. Therefore, at the end of data extraction and conversion, the faa, fna, and embl files were obtained, as well as a csv file containing information about gene positions, gene names, gc content of each gene, products, and locus tags.

### Step 2: GC content variability

The deviation in the GC content at specific intervals of the genome is considered a consolidated signature of putative exogenous regions^8,43^, making them an ideal starting point for predicting genomic islands. To detect compositional anomalies, the GC content was calculated for each gene and compared to the genome-wide average, allowing identification of regions with GC content bias. The ffn and fna files were used to calculate the deviation in GC content. Firstly, the GC deviation is calculated considering the formula below:

$$GC\sigma = \sqrt {\frac{{\sum \left( {gc_{i} - GC} \right)^{2} }}{n}}$$

where *gc* represents the GC content of each gene in the genome, *GC* represents the GC content of the whole genome, and *n* represents the total number of genes in the genome. Right after the GC deviation is calculated using the formula below:

$$\Delta gc = \frac{{\left| {\left. {GC - gc} \right|} \right.}}{GC\sigma }$$

### Step 3: codon usage bias

Members of the same bacterial species tend to share the same codon usage rates^24,44^. Codon usage bias is a genetic characteristic that has been studied for its ability to easily distinguish exogenous elements from endogenous genomic entities^45^. This is because different genes in the same genome tend to maintain a constant rate of usage of the same codon to encode the same amino acid, which is advantageous for tRNA bioavailability. In the GIPSy2 pipeline, the Colombo/Sigi-HMM program^41^ was used to predict the deviations in codon usage for each gene nucleotide sequence. This program uses its own databases and a Hidden Markov Model to determine the codon usage rate of each amino acid encoded by the genome and for each gene. The main result obtained was a list containing genes that show deviations in codon usage and are assumed to be putative horizontally transferred genes.

### Step 4: Searching for mobile elements

In addition to the new version of GIPSy, a database of elements related to gene mobility has been updated. The new version uses all X categories from the Clusters of Orthologous Groups (COG) database^46^. This category includes proteins related to the mobilome such as integrons, transposases, and phages. At this stage of the analysis, the proteomes extracted from each genome were aligned using DIAMOND against the database, hereafter named the mobilome, and the presence of an element was configured by alignment with a maximum e-value of 5^e-06^.

### Step 5: searching for specific factors

To categorize the predicted islands, the factors present in each DNA segment were compared. Specific databases were used for each island class considered in the analysis. This comparison was based on the predicted proteome of each strain extracted from the GBK file used as input. Alignment of each proteome was performed using the DIAMOND aligner with a maximum e-value of 5e^-06^.

Specific databases dedicated to specific functions are selected by the user at the beginning of the analysis. To search for resistance factors, the Comprehensive Antibiotic Resistance Database (CARD) version 4.0.1 is used, which contains a robust dataset of genes specifically related to the microbial resistance phenotype that are present in chromosomes or plasmids^47,48^.

Virulence factors were predicted based on comparisons with the Virulence Factors Database (VFDB)^49^, which contains a robust set of genes related to bacterial virulence, pathogenicity, and infectivity.

Symbiosis factors were predicted based on comparisons with the NodMutDB database^50^, which contains genes involved in the process of bacterial symbiosis in plants and survival in soil, as well as genes related to environmental recycling processes, such as nitrogen fixation.

Finally, a database related to genes involved in general bacterial metabolic processes has been established, including the COG categories C (energy production and conversion), E (amino acid transport and metabolism), F (nucleotide transport and metabolism), G (carbohydrate transport and metabolism), H (coenzyme transport and metabolism), I (lipid transport and metabolism), P (inorganic ion transport and metabolism), Q (secondary metabolite biosynthesis, transport and catabolism)^46^. The resulting sub-datasets were concatenated and their redundancy was removed using the seqkit tool^51^.

### Step 6: reciprocal alignments

Genomic islands are fragments which do not belong to the core genome of a species. Therefore, the reliability of the analysis increases if a putative island is not found in a taxonomically associated strain but has a divergent specific phenotype^16^. To make this inference, the protein content of the query lineage was compared to that of the subject lineage using multiple alignments of their proteomes. The results of this alignment were added to the generated temporary file, and pseudo-synteny was evaluated considering the genes neighborhood and based on the locus tag order of both genomes. The proportion of proteins missing from the subject’s genome was calculated by generating values ranging from 0 to 1. The closer the proportion value is to 1, the greater the likelihood that the predicted region is a genomic island.

### Step 7: searching for tRNAs

The GIs are characterised by the presence of tRNA clusters on their flanks. This can be explained by repetitive patterns in the coding sequences of tRNAs, which facilitate their recognition by restriction enzymes and integrases. Considering this pattern, GIPSy 2 searches for tRNAs using multiple alignments against the tRNADB-CE database^52^ with a specific portion of the bacterial genome.

### Step 8: merging results

The results obtained in the previous steps were grouped into a. csv file, the order of which corresponded to the arrangement of genes in the query genome file. This makes interpretation intuitive as it facilitates integration between the genomic data and the results obtained, highlighting regions that may be skewed.

### Step 9: genomic island (GI) prediction

The starting point for island prediction in the last step was to group the regions with a GC content bias or absence in the reference. This is accomplished by finding the first gene of interest and concatenating the positions of the subsequent genes that also have the same deviation until no further bias is found. By default, the search for biased genes was performed up to 10 kb from the last gene added to the putative mobile region. If no gene was found within this range, the region was closed to the position of the last gene showing GC content deviation or absence in the reference genome in the sequence fragment.

With the regions stored, the information obtained in steps 3–7 was added to each putative island region to form a matrix with previously predicted factor ratio data. The numerical matrix is subjected to one of six major statistical methods selected by the user: (I) hard, (II) Fisher, (III) principal component analysis (PCA), (IV) singular value decomposition (SVD), (V) logistic regression, and (VI) support vector machine (SVM) classifier. Supervised learning models (e.g., SVM and logistic regression) were trained using feature matrices derived from intermediate outputs of the pipeline. Labels were assigned based on the presence or absence of BEIPs, and the models were used as components of the integrated prediction framework rather than as independent classifiers.

*Hard method.* The hard method logic is the least permissive of the new methods in this version of GIPSy. It considers a region to be a putative genomic island if it has all the required BEIPs in absolute terms, and the proportion of those BEIPs in the putative region is higher than the whole genomic content. In this case, the final score is a binary variable, where 0 indicates that the region is not an island, and 1 indicates that the region is a putative island. This is not a statistical test but a rule-based comparison using fixed thresholds acting as a strict filter. Because of that, it is possible to say that this is a deterministic assumption. Owing to the variable and plastic nature of the genome, pattern-following cannot be guaranteed. Therefore, the construction of genomic islands in certain strains is achieved using unusual methods, such as the absence of mobile-associated elements within the islands or the absence of tRNAs flanking these regions. In these cases, absolute counting of BEIPs is not feasible.

*Fisher method.* The Fisher method is the most similar to the logic of the first version of GIPSy. In this approach, a region is considered a putative island if the proportion of BEIPs in the region is higher than the proportion of BEIPs in the rest of the genome. A contingency table was constructed for each type of BEIP and putative region. To construct a contingency table that was subjected to Fisher’s exact test^53^, the following were considered: (A) number of BEIPs within the region, (B) number of putative elements unrelated to islands within the region, (C) number of a given BEIP in the genome, and (D) number of putative elements unrelated to islands in the genome. For the statistical model, the alternative hypothesis was that the ratio of the proportion of BEIPs within the putative region is greater than 1. As we used Fisher’s exact test for multiple paired comparisons, we adjusted our initial significance value of 0.05 to 0.05 divided by the number of comparisons, which we rounded to 0.01 for each test. As a result, the p-value obtained is evaluated, and if the result is equal to or below 0.01, a positive Boolean value is associated with the element analysed for the region in question. The final result is a vector containing the scores determined by the average of the binary values assigned to each region, which can therefore vary between 0 and 1, where 1 indicates that all the BEIPs analysed are in greater proportion within the island, and 0 indicates that the proportion of the island content is equal to or less than the proportion of the rest of the genome.

*Principal component analysis (PCA) method.* Principal component analysis aims to reduce the dimensionality of a data set by summarizing the variance of its initial variables into coordinates of equal or very close variance^54,55^. Ultimately, this vectorisation aims to generate a new hypothetical set of variables that stores most of the variations in the original dataset. This was achieved by constructing a matrix with the previously grouped putative regions as elements and the BEIPs within each region as features. This matrix is then dimensionalized using the PCA method, and the values of the components that recover most of the variation in the original dataset are stored in the form of a simple vector. The vector values were then normalised using the max–min method according to the formula $score =\frac{{x}_{i} - {x}_{(min)}}{{x}_{(min)} - {x}_{(max)}}$, producing a final score for each putative region. Therefore, higher score values are expected to represent genomic islands, since the variance of BEIPs in exogenous regions tends to be significantly higher than the variance of these same elements in endogenous regions.

*Singular value decomposition (SVD) method.* Singular value decomposition is another dimensionality reduction method that aims to reduce the complexity of a matrix into a vector containing the patterns of each element constituting the original matrix^56^. The logic behind the use of SVD is the same as that of PCA. The variance of the elements was expected to be sufficient to generate higher SVD eigenvalues in the putative genomic island regions. Therefore, SVD was applied to a matrix containing the proportion of BEIPs in each region extracted from the genome, to obtain a vector containing a single predicted value for each region. This value represents an orthogonal coordinate denoting an equivalent principal component. After max–min normalisation, the coordinate values are called scores because they summarise a large part of the variance in the BEIP values within each putative region.

*Support vector machine (SVM) classifier method.* SVM is a statistical and computational method applied to solve regression and classification problems. The model aims to construct an n-1 dimensional hyperplane that separates the elements into two different classes with the largest possible margin^57^. In the case of GIPSy2, this method was used to determine whether a region was a putative island in the binary form. A matrix containing vectors representing each BEIP value related to all the putative regions was used as the input. The response variable used in this method is a vector that includes binary values for the presence or absence of a region in a reference genome. By default, a region is considered absent when it has at least 60% gene absence compared to a reference. The value can also be set by the user. The result is a binary vector that classifies the regions, and a score value that represents the average accuracy of the model in correctly separating the dataset into two categories based exclusively on the response variable. The final score reflects the model’s ability to separate these classes rather than an independent measure of predictive performance.

*Logistic regression method.* To work properly, logistic regression requires a response variable composed of binary values that represent expected outcomes^58–60^. For GIPSy2 predictions, these values cannot be given precisely because they require prior knowledge of the genomic data of the organisms used as queries and subjects. To address this issue, historical data collection was selected to provide information on the presence and location of genomic islands from the most diverse origins, in the absence of a well-established reference region. Generally, genomic islands are not present in all lineages of a species; therefore, the absence of a region in a lineage with a contrasting phenotype can be a strong indication of the reliability of the presence of an island in a given region. Under such circumstances, a vector containing binary values of the absence of a segment lacking a reference within each previously segregated putative region is used to predict the coordinate values generated by logistic regression. This assumes that the variability of the explanatory variables is sufficient to separate putative regions orthogonally from the artefact regions. However, the logistic regression model relies on internally defined response variables and should be interpreted as a complementary classification approach rather than an independently validated predictive model.

*Additional consensus approach.* An optional consensus filtering procedure was implemented to increase confidence in GI predictions. When selected by the user, only regions predicted by at least three independent methods are retained for downstream reporting. This threshold was defined as a pragmatic criterion to integrate evidence from multiple methods while reducing method-specific bias. The consensus level is user-configurable, allowing adjustment according to the desired balance between sensitivity and specificity. GI prediction algorithms evaluate distinct genomic signals, such as compositional deviations, atypical codon usage, mobility-related genes, and insertion loci. Additionally, they might operate under different statistical and evolutionary assumptions about genome organization. Because these assumptions may not be uniformly satisfied across genomes, an individual approach can produce dataset-dependent false-positive or false-negative results. Requiring agreement among multiple predictors integrates orthogonal evidence and functions as a confidence filter applied during the final consolidation step. As this procedure is user-configurable, the workflow preserves analytical flexibility, allowing adjustment of prediction stringency according to the characteristics of the dataset and the objectives of the analysis. However, the choice of consensus threshold directly impacts prediction stringency, and different thresholds may be more appropriate depending on the characteristics of the dataset and the objectives of the analysis.

### Comparative analysis

Comparative analyses were based on a comparison of the results obtained using GIPSy1 and GIPSy2. For this purpose, query and subject genomes were selected for each analysis with respect to the accessions described in Table 2. As the efficiency of the first version of GIPSy was previously verified, islands predicted using the original pipeline were used as a reference for developing the new version. The genomes were selected to match the dataset used in the publication of the first version of GIPSy. In addition, they were retained due to the extent of research conducted on them and their description in the literature. Moreover, the selected model organisms represent diverse ecological niches, reducing the likelihood of bias due to shared genomic features among closely related bacteria.

**Table 2:** Genomes selected for comparative analysis.

Query			Subject			Factor analysed
Species	Strain	Accession number	Species	Strain	Accession number	
*Acinetobacter baumannii*	AYE	GCA_000069245.1	*Acinetobacter baumannii*	SDF	GCA_000069205.1	Resistance
*Burkholderia pseudomallei*	K96243	GCA_000959285.1	*Burkholderia pseudomallei*	668	GCA_000015905.1	Metabolic
*Escherichia coli*	CFT073	GCA_021559855.1	*Escherichia coli*	K-12	GCA_009832885.1	Virulence
Mesorhizobium japonicum*	MAFF 303,099	GCA_000009625.1	*Mesorhizobium sp.*	BNC1	GCF_000014245.1	Symbiosis
			*Mesorhizobium loti*	TONO	GCF_002356515.1	
			*Mesorhizobium loti*	R88b	GCF_013170845.1	
			*Mesorhizobium japonicum*	R7Astar	GCF_012913645.1	

* Species used for the multiple reference analysis.

All genomes listed in Table 2 were annotated using the PROKKA pipeline (20). BLAST ring plots were generated using the BRIG software^61^, and comparative genomes were obtained from the NCBI database (Supplementary table 9). In view of the methodological changes in the new version, the islands predicted by GIPSy2 were considered to overlap with those predicted by GIPSy1 if there was a minimum coverage of 75% between the second and first versions of the prediction. The islands of interest were manually evaluated using the Proksee server^62^. A Proksee session is a structured file used for visualization in the Proksee platform, enabling interactive inspection of predicted genomic islands within circular genome maps.

### Performance analysis

For an empirical complexity analysis, 300 genomes of *E. coli* were obtained from the NCBI database (see Supplementary table 10) and used as a query against *E. coli* K-12 genome. The analysis was performed on a server composed of 64 GB of RAM, 1 TB SSD, and an AMD Ryzen 9 5900 12 processor.

Statistical comparative analysis was based on previous validated results of in vitro genomic island identification of *E. coli* CFT073 published by Lloyd et al.^63^, which employed comparative genomic hybridization with microarrays to identify variable genomic regions hereby called “plastic spots” among *E. coli* strains focusing on the CFT073. This study revealed a wealth of plastic spots, with a focus on regions larger than 30 kb. Genes found simultaneously inside a predicted and validated genomic island were considered true positives for accuracy, sensitivity, and specificity measurements. The analysis was performed several times, adjusting the parameters required for island prediction, such as the minimum length of a region to be considered an island and the minimum number of bases between two islands. A precision test was performed for each run. To ensure the statistical accuracy of our approach, we also considered the results of the IslandViewer analysis^64^ for the same strain for comparative purposes. IslandViewer was selected as an external benchmark due to its widespread use and its integration of multiple genomic island prediction methods, encompassing both compositional and comparative approaches^14,17,65^. This makes it a representative reference for evaluating genomic island prediction performance. However, we acknowledge that the use of a single external tool limits the scope of the comparison, and future studies including a broader range of standalone predictors would provide a more comprehensive benchmarking framework.

Due to the limited number of experimentally validated genomic islands available, additional regions predicted by GIPSy1 were incorporated solely to expand the set of candidate genomic islands for exploratory and comparative analyses. These regions were not used as ground truth in the calculation of performance metrics.

All methods were evaluated using their default parameterization to ensure a consistent basis for comparison. For GIPSy2, an additional ad hoc parameterization was explored to assess the impact of user-defined settings on performance, but this configuration was not used as the primary benchmark for comparison. The ad hoc parameterization consisted of adjusting the minimum length of a genomic island to 26 kbp and the minimum step size between distinct genomic islands to 26 kbp. These settings were selected to align with the size constraints of the reference dataset used for benchmarking, which is limited in its ability to detect smaller genomic islands. As a result, this configuration is expected to influence performance metrics, particularly by reducing apparent false positives, and should therefore be interpreted with caution.

## Supplementary Information

Below is the link to the electronic supplementary material.

Supplementary Material 1

Supplementary Material 2

Supplementary Material 3

Supplementary Material 4

Supplementary Material 5

Supplementary Material 6

Supplementary Material 7

Supplementary Material 8

Supplementary Material 9

Supplementary Material 10

## Data Availability

The datasets were derived from sources in the public domain and its description is available on online supplementary table 9 and supplementary table 10. The data underlying this article and its support materials are available in the Zenodo repository at 10.5281/zenodo.10214337. The pre-compiled versions of GIPSy2 are available in the Zenodo repository at 10.5281/zenodo.10222587.

## References

[1] Land, M. et al. Insights from 20 years of bacterial genome sequencing. *Funct. Integr. Genomics* **15**, 141–161 (2015). 25722247 10.1007/s10142-015-0433-4 PMC4361730

[2] Kumar, S. & Dudley, J. Bioinformatics software for biologists in the genomics era. *Bioinformatics* **23**, 1713–1717 (2007). 17485425 10.1093/bioinformatics/btm239

[3] Hacker, J. & Kaper, J. B. Pathogenicity islands and the evolution of microbes. *Annu. Rev. Microbiol.* **54**, 641–679 (2000). 11018140 10.1146/annurev.micro.54.1.641

[4] Juhas, M. et al. Genomic islands: Tools of bacterial horizontal gene transfer and evolution. *FEMS Microbiol. Rev.* **33**, 376–393 (2009). 19178566 10.1111/j.1574-6976.2008.00136.x PMC2704930

[5] Yoon, S. H., Park, Y.-K. & Kim, J. F. PAIDB v2.0: Exploration and analysis of pathogenicity and resistance islands. *Nucleic Acids Res.* **43**, D624–D630 (2015). 25336619 10.1093/nar/gku985 PMC4384037

[6] Finan, T. M. Evolving insights: Symbiosis islands and horizontal gene transfer. *J. Bacteriol.* **184**, 2855–2856 (2002). 12003923 10.1128/JB.184.11.2855-2856.2002 PMC135049

[7] Li, W. & Wang, A. Genomic islands mediate environmental adaptation and the spread of antibiotic resistance in multiresistant *Enterococci* - Evidence from genomic sequences. *BMC Microbiol.* **21**, 55 (2021). 33602143 10.1186/s12866-021-02114-4 PMC7893910

[8] Zhang, R., Ou, H.-Y., Gao, F. & Luo, H. Identification of horizontally-transferred genomic islands and genome segmentation points by using the GC profile method. *Curr. Genomics* **15**, 113–121 (2014). 24822029 10.2174/1389202915999140328163125 PMC4009839

[9] Furlan, J. P. R., Savazzi, E. A. & Stehling, E. G. Genomic insights into multidrug-resistant and hypervirulent *Klebsiella pneumoniae* co-harboring metal resistance genes in aquatic environments. *Ecotoxicol. Environ. Saf.* **201**, 110782 (2020). 32497817 10.1016/j.ecoenv.2020.110782

[10] Arnold, B. J., Huang, I.-T. & Hanage, W. P. Horizontal gene transfer and adaptive evolution in bacteria. *Nat. Rev. Microbiol.* **20**, 206–218 (2022). 34773098 10.1038/s41579-021-00650-4

[11] Hacker, J. & Carniel, E. Ecological fitness, genomic islands and bacterial pathogenicity. *EMBO Rep.* **2**, 376–381 (2001). 11375927 10.1093/embo-reports/kve097 PMC1083891

[12] Fernández-Gómez, B. et al. Patterns and architecture of genomic islands in marine bacteria. *BMC Genomics* **13**, 347 (2012). 22839777 10.1186/1471-2164-13-347 PMC3478194

[13] Lu, B. & Leong, H. W. Computational methods for predicting genomic islands in microbial genomes. *Comput. Struct. Biotechnol. J.* **14**, 200–206 (2016). 27293536 10.1016/j.csbj.2016.05.001 PMC4887561

[14] da Silva Filho, A. C. *et al.* Comparative Analysis of Genomic Island Prediction Tools. *Front. Gene.* **9**, (2018). 10.3389/fgene.2018.00619 PMC6315130 30631340

[15] Bertelli, C., Tilley, K. E. & Brinkman, F. S. L. Microbial genomic island discovery, visualization and analysis. *Brief. Bioinform.* **20**, 1685–1698 (2019). 29868902 10.1093/bib/bby042 PMC6917214

[16] Soares, S. C. et al. GIPSy: Genomic island prediction software. *J. Biotechnol.* **232**, 2–11 (2016). 26376473 10.1016/j.jbiotec.2015.09.008

[17] Chakraborty, J., Roy, R. P., Chatterjee, R. & Chaudhuri, P. Performance assessment of genomic island prediction tools with an improved version of *Design-Island*. *Comput. Biol. Chem.* **98**, 107698 (2022). 35597186 10.1016/j.compbiolchem.2022.107698

[18] Sahl, J. W. et al. Genomic comparison of multi-drug resistant invasive and colonizing *Acinetobacter baumannii* isolated from diverse human body sites reveals genomic plasticity. *BMC Genomics* **12**, 291 (2011). 21639920 10.1186/1471-2164-12-291 PMC3126785

[19] Fournier, P.-E. et al. Comparative genomics of multidrug resistance in *Acinetobacter baumannii*. *PLoS Genet.* **2**, e7 (2006). 16415984 10.1371/journal.pgen.0020007 PMC1326220

[20] Semail, N. et al. Genomic dataset of eighteen *Burkholderia pseudomallei* strains isolated from clinical and environmental settings in Malaysia. *Curr. Res. Microb. Sci.* **8**, 100397 (2025). 40547377 10.1016/j.crmicr.2025.100397 PMC12181971

[21] Sim, S. H. et al. The core and accessory genomes of *Burkholderia pseudomallei*: Implications for human melioidosis. *PLoS Pathog.* **4**, e1000178 (2008). 18927621 10.1371/journal.ppat.1000178 PMC2564834

[22] Schneider, M. V. & Jimenez, R. C. Bioinformatics: Scalability, capabilities and training in the data-driven era. *Brief. Bioinform.* **20**, 735–736 (2019). 31100108 10.1093/bib/bbz053

[23] Wratten, L., Wilm, A. & Göke, J. Reproducible, scalable, and shareable analysis pipelines with bioinformatics workflow managers. *Nat. Methods* **18**, 1161–1168 (2021). 34556866 10.1038/s41592-021-01254-9

[24] Sharp, P. M., Bailes, E., Grocock, R. J., Peden, J. F. & Sockett, R. E. Variation in the strength of selected codon usage bias among bacteria. *Nucleic Acids Res.* **33**, 1141–1153 (2005). 15728743 10.1093/nar/gki242 PMC549432

[25] Zhang, R. & Zhang, C.-T. A systematic method to identify genomic islands and its applications in analyzing the genomes of *Corynebacterium glutamicum* and *Vibrio vulnificus* CMCP6 chromosome I. *Bioinformatics* **20**, 612–622 (2004). 15033867 10.1093/bioinformatics/btg453

[26] Bohlin, J. et al. Analysis of intra-genomic GC content homogeneity within prokaryotes. *BMC Genomics* **11**, 464 (2010). 20691090 10.1186/1471-2164-11-464 PMC3091660

[27] Langille, M. G. I., Hsiao, W. W. L. & Brinkman, F. S. L. Detecting genomic islands using bioinformatics approaches. *Nat. Rev. Microbiol.* **8**, 373–382 (2010). 20395967 10.1038/nrmicro2350

[28] Vallenet, D. et al. Comparative analysis of Acinetobacters: Three genomes for three lifestyles. *PLoS ONE* **3**, e1805 (2008). 18350144 10.1371/journal.pone.0001805 PMC2265553

[29] Vernikos, G. S. & Parkhill, J. Resolving the structural features of genomic islands: A machine learning approach. *Genome Res.* **18**, 331–342 (2008). 18071028 10.1101/gr.7004508 PMC2203631

[30] Holden, M. T. G. et al. Genomic plasticity of the causative agent of melioidosis, *Burkholderia pseudomallei*. *Proc. Natl. Acad. Sci. U. S. A.* **101**, 14240–14245 (2004). 15377794 10.1073/pnas.0403302101 PMC521101

[31] Lloyd, A. L., Henderson, T. A., Vigil, P. D. & Mobley, H. L. T. Genomic islands of uropathogenic *Escherichia coli* contribute to virulence. *J. Bacteriol.* **191**, 3469–3481 (2009). 19329634 10.1128/JB.01717-08 PMC2681901

[32] Luo, C., Hu, G.-Q. & Zhu, H. Genome reannotation of *Escherichia coli* CFT073 with new insights into virulence. *BMC Genomics* **10**, 552 (2009). 19930606 10.1186/1471-2164-10-552 PMC2785843

[33] Merkl, R. SIGI: Score-based identification of genomic islands. *BMC Bioinform.* **5**, 22 (2004). 10.1186/1471-2105-5-22 PMC394314 15113412

[34] Liu, H. & Zhu, J. Analysis of the 3′ ends of tRNA as the cause of insertion sites of foreign DNA in *Prochlorococcus*. *J. Zhejiang Univ. Sci. B* **11**, 708–718 (2010). 20803775 10.1631/jzus.B0900417 PMC2932881

[35] Beumer, A. & Robinson, J. B. A broad-host-range, generalized transducing phage (SN-T) acquires 16S rRNA genes from different genera of bacteria. *Appl. Environ. Microbiol.* **71**, 8301–8304 (2005). 16332816 10.1128/AEM.71.12.8301-8304.2005 PMC1317401

[36] Espejo, R. T. & Plaza, N. Multiple ribosomal RNA operons in bacteria; Their concerted evolution and potential consequences on the rate of evolution of their 16S rRNA. *Front. Microbiol.* **9**, (2018). 10.3389/fmicb.2018.01232 PMC6002687 29937760

[37] Uchiumi, T. et al. Expression Islands clustered on the symbiosis island of the mesorhizobium loti genome. *J. Bacteriol.* **186**, 2439–2448 (2004). 15060047 10.1128/JB.186.8.2439-2448.2004 PMC412173

[38] Monaghan, T. F. et al. Foundational statistical principles in medical research: Sensitivity, specificity, positive predictive value, and negative predictive value. *Medicina (Kaunas)* **57**, 503 (2021). 34065637 10.3390/medicina57050503 PMC8156826

[39] Van Rossum, G. & Drake, F. L. *Python 3 Reference Manual*. (2009).

[40] Buchfink, B., Xie, C. & Huson, D. H. Fast and sensitive protein alignment using DIAMOND. *Nat. Methods* **12**, 59–60 (2015). 25402007 10.1038/nmeth.3176

[41] Waack, S. et al. Score-based prediction of genomic islands in prokaryotic genomes using hidden Markov models. *BMC Bioinform.* **7**, 142 (2006). 10.1186/1471-2105-7-142 PMC1489950 16542435

[42] Seemann, T. Prokka: Rapid prokaryotic genome annotation. *Bioinformatics* **30**, 2068–2069 (2014). 24642063 10.1093/bioinformatics/btu153

[43] Gao, F. & Zhang, C.-T. GC-Profile: A web-based tool for visualizing and analyzing the variation of GC content in genomic sequences. *Nucleic Acids Res.* **34**, W686–W691 (2006). 16845098 10.1093/nar/gkl040 PMC1538862

[44] Bolotin, E. & Hershberg, R. Horizontally Acquired Genes Are Often Shared between Closely Related Bacterial Species. *Front. Microbiol.* **8**, (2017). 10.3389/fmicb.2017.01536 PMC5575156 28890711

[45] Bailly-Bechet, M., Danchin, A., Iqbal, M., Marsili, M. & Vergassola, M. Codon usage domains over bacterial chromosomes. *PLoS Comput. Biol.* **2**, e37 (2006). 16683018 10.1371/journal.pcbi.0020037 PMC1447655

[46] Tatusov, R. L. et al. The COG database: An updated version includes eukaryotes. *BMC Bioinform.* **4**, 41 (2003). 10.1186/1471-2105-4-41 PMC222959 12969510

[47] Alcock, B. P. et al. CARD 2023: Expanded curation, support for machine learning, and resistome prediction at the comprehensive antibiotic resistance database. *Nucleic Acids Res.* **51**, D690–D699 (2023). 36263822 10.1093/nar/gkac920 PMC9825576

[48] Alcock, B. P. et al. CARD 2020: Antibiotic resistome surveillance with the Comprehensive Antibiotic Resistance Database. *Nucleic Acids Res.* **48**, D517–D525 (2020). 31665441 10.1093/nar/gkz935 PMC7145624

[49] Liu, B., Zheng, D., Zhou, S., Chen, L. & Yang, J. VFDB 2022: A general classification scheme for bacterial virulence factors. *Nucleic Acids Res.* **50**, D912–D917 (2022). 34850947 10.1093/nar/gkab1107 PMC8728188

[50] Mao, C., Qiu, J., Wang, C., Charles, T. C. & Sobral, B. W. S. NodMutDB: A database for genes and mutants involved in symbiosis. *Bioinformatics* **21**, 2927–2929 (2005). 15817696 10.1093/bioinformatics/bti427

[51] Shen, W., Le, S., Li, Y. & Hu, F. SeqKit: A cross-platform and ultrafast toolkit for FASTA/Q file manipulation. *PLoS ONE* **11**, e0163962 (2016). 27706213 10.1371/journal.pone.0163962 PMC5051824

[52] Abe, T. *et al.* tRNADB-CE: tRNA gene database well-timed in the era of big sequence data. *Frontiers in Genetics* **5**, (2014). 10.3389/fgene.2014.00114 PMC4013482 24822057

[53] Kim, H.-Y. Statistical notes for clinical researchers: Chi-squared test and Fisher’s exact test. *Restor. Dent. Endod.* **42**, 152–155 (2017). 28503482 10.5395/rde.2017.42.2.152 PMC5426219

[54] Hasan, B. M. S. & Abdulazeez, A. M. A review of principal component analysis algorithm for dimensionality reduction. *J. Soft Comput. Data Min.* **2**, 20–30 (2021).

[55] Ringnér, M. What is principal component analysis?. *Nat. Biotechnol.* **26**, 303–304 (2008). 18327243 10.1038/nbt0308-303

[56] Gerbrands, J. J. On the relationships between SVD, KLT and PCA. *Pattern Recogn.* **14**, 375–381 (1981).

[57] Yue, S., Li, P. & Hao, P. SVM classification:Its contents and challenges. *Appl. Math.-A J. Chin. Univ.* **18**, 332–342 (2003).

[58] Magder, L. S. & Hughes, J. P. Logistic regression when the outcome is measured with uncertainty. *Am. J. Epidemiol.* **146**, 195–203 (1997). 9230782 10.1093/oxfordjournals.aje.a009251

[59] Wright, R. E. Logistic regression. In *Reading and Understanding Multivariate Statistics* 217–244 (American Psychological Association, 1995).

[60] LaValley, M. P. Logistic regression. *Circulation* **117**, 2395–2399 (2008). 18458181 10.1161/CIRCULATIONAHA.106.682658

[61] Alikhan, N.-F., Petty, N. K., Ben Zakour, N. L. & Beatson, S. A. BLAST ring image generator (BRIG): Simple prokaryote genome comparisons. *BMC Genomics* **12**, 402 (2011). 21824423 10.1186/1471-2164-12-402 PMC3163573

[62] Grant, J. R. et al. Proksee: In-depth characterization and visualization of bacterial genomes. *Nucleic Acids Res.* **51**, W484–W492 (2023). 37140037 10.1093/nar/gkad326 PMC10320063

[63] Lloyd, A. L., Rasko, D. A. & Mobley, H. L. T. Defining genomic islands and uropathogen-specific genes in uropathogenic Escherichia coli. *J Bacteriol* **189**, 3532–3546 (2007). 17351047 10.1128/JB.01744-06 PMC1855899

[64] Bertelli, C. et al. IslandViewer 4: Expanded prediction of genomic islands for larger-scale datasets. *Nucleic Acids Res.* **45**, W30–W35 (2017). 28472413 10.1093/nar/gkx343 PMC5570257

[65] Bertelli, C. et al. Enabling genomic island prediction and comparison in multiple genomes to investigate bacterial evolution and outbreaks. *Microb. Genomics* **8**, mgen000818 (2022). 10.1099/mgen.0.000818 PMC9465072 35584003

